# Dissociative Experiences Mediate the Relationship Between Traumatic Life Events and Types of Skin Picking. Findings From Non-clinical Sample

**DOI:** 10.3389/fpsyt.2021.698543

**Published:** 2021-07-19

**Authors:** Joanna Kłosowska, Rachela Antosz-Rekucka, Alina Kałużna-Wielobób, Katarzyna Prochwicz

**Affiliations:** ^1^Institute of Psychology, Jagiellonian University, Krakow, Poland; ^2^Institute of Psychology, Pedagogical University, Krakow, Poland

**Keywords:** skin picking, automatic skin picking, focused skin picking, trauma, dissociation, mediation

## Abstract

**Aim:** Skin-picking (excoriation) disorder is considered as a form of maladaptive coping methods used by individuals who have difficulties in applying more adaptive strategies. Skin-picking development has been suggested to be preceded by traumatic life events. Dissociative symptoms have been reported as experienced by skin-picking sufferers during picking episodes. The purpose of the study was to examine whether the link between trauma and automatic type of skin-picking is mediated by the frequency of dissociative experiences, and whether the COVID-19 pandemic conditions have changed this relationship in any way.

**Methods:** The study sample consisted of 594 adults (76% women) aged from 18 to 60. Traumatic life events, dissociative experiences, and types of skin-picking (focused vs. automatic) were assessed with self-report questionnaires. Mediation analyses and multigroup path analyses were carried out.

**Results:** Dissociative experiences partially mediated the link between traumatic events and both types of skin-picking. The model was robust considering the conditions in which survey was filled out (pre-pandemic vs. pandemic).

**Conclusions:** Traumatic life events and dissociative experiences are associated with both automatic and focused skin-picking regardless of pandemic conditions. Further studies are needed to understand mechanisms underlying the relationship between dissociation and skin-picking styles.

## Introduction

Excoriation disorder, also referred to as skin-picking disorder (SPD) or dermatillomania is characterised by recurrent and excessive picking of the skin (healthy areas or/and dermatological irregularities) which cannot be better explained by the presence of other medical or psychiatric conditions or substance use. Affected individuals take repeated attempts to reduce or stop harming the skin which are usually ineffective. Overdone picking performed for some time results in noticeable skin damage and/or significant impairment in important areas of psychosocial functioning (1).

Picking behaviours include at least two different subtypes whose frequency varies over disorder course and depends on trigger factors. Some acts of picking are performed habitually and without awareness, whereas others are taken intentionally in order to decrease unpleasant tension or to get rid of skin irregularities. Typically, pathological skin manipulation is unconscious at the disorder onset, but after a certain period of time it becomes more aware (2, 3). Although most sufferers report both styles of picking, one style is usually more prominent than the other (4). The presence of these two picking subtypes, labelled “automatic” and “focused” respectively, was confirmed in prior empirical studies (e.g., (5–9)].

Picking behaviours are not restricted to clinically relevant symptoms, but represent a phenomenon of different intensity (10–12). It is estimated that pathological excoriation occurs in 1.4–5.4% of adults (13, 14) whereas the transient, subclinical manifestations of the disorder affect 62.7% of the general population (11) and even 91.7% of university sample (10).

In the recent edition of the Diagnostic Manual of Mental Disorders (DSM 5) excoriation has been listed within the broader category of Obsessive-Compulsive and Related Disorders (DSM-5) among other psychiatric conditions recognised as sharing phenomenological similarities (1). Apart from highlighting phenomenological characteristics, the clustering of OCD-spectrum disorders in one category directs attention towards etiological factors they have in common.

Substantial studies indicated that OCD-spectrum disorders, trichotillomania in particular, develop to modulate aversive internal states (4, 15–21). From this point of view, repetitive behaviours such as hair-pulling or skin-picking may be considered as emotion regulation strategies. These strategies, however, are perceived as maladaptive: although they provide immediate relief, in the long run they exacerbate psychopathological symptoms (e.g., depression, anxiety, guilt, negative self-image, low self-esteem). Such strategies are usually chosen when stress is so overwhelming that it is beyond the individual's ability to deal with, as is the case with trauma exposure.

Traumatic life events have been demonstrated to have far-reaching psychological sequelae, among which the development of post-traumatic stress disorder (PTSD) has gained the greatest interest. However, the predictive role of trauma has also been reported in studies concerning other forms of psychopathology [e.g., (22–27)]. What is more, trauma experience was found to impede an individual's coping abilities (28) and decreases tolerance to subsequent stressors (29–31).

Dissociative symptoms are an important clinical consequence of trauma exposure which increases the risk of further development of psychiatric conditions [e.g., (32–35)]. Dissociation as a result of trauma can be defined as detachment from ongoing reality and loss of normal integration between thoughts, feelings and experiences into the stream of consciousness and memory (36). Psychoanalytic authors [e.g., (37)] view dissociation as a defence strategy against trauma involving disengagement of the mind capacity to perceive unbearable realty. This process allows person to preserve sense of integrity and protects his/her against destabilisation of experience of selfhood. Although generalised dissociation is considered to be maladaptive, in individuals struggling with traumatic life events it may serve as a protective mechanism that allows them to detach psychologically from the events that are too overwhelming to cope with (38).

An increasing number of studies has indicated that traumatic life events are likely to influence the pathways to a variety of OCD-spectrum disorders. Trauma exposure has been found to precede obsessive-compulsive disorder (39–41), trichotillomania (40), body dysmorphic disorder (42) and hoarding symptom (43). Preliminarily studies have also shown the link between childhood trauma and skin-picking (44, 45). There is also evidence, that the probability for potential traumatic events to foster psychopathology depends on the type of traumatization (e.g., emotional, physical, sexual) and that interpersonal traumas, primarily sexual abuse, may contribute to symptoms development more severely than non-interpersonal ones (46, 47). Moreover, among the OCD-spectrum disorders skin-picking and trichotillomania have been shown to involve varying degrees of consciousness (9). What is more, previous research appears to support the notion that both picking and pulling behaviours display dissociative features, that is, appear to occur without full awareness: one-third of individuals with skin-picking report a feeling of trance or a feeling of being mesmerised during the act of picking (4, 48), whereas over one-fifth of patients suffering from trichotillomania experience depersonalisation when pulling hair (49). It supports the assumption that dissociation may be a mediator in the relationship between trauma and skin-picking/hair pulling, when performed in order to regulate aversive internal states.

### Aims and Hypothesis

In the current study we examined the relationship between trauma, dissociation and skin-picking behaviours. In particular, we tested whether the presence of dissociative symptoms mediates the relationship between traumatic life events and automatic style of picking. Since the experience of chronic interpersonal trauma in childhood is assumed to disrupt the development of adaptive emotion regulation and decrease the ability to apply effective coping methods (50, 51), we assumed that trauma may be a factor favouring the development of skin-picking as a regulation strategy. Additionally, since symptoms of dissociation are listed among important trauma consequences we assumed that trauma predicts automatic skin-picking, that is, picking style performed mostly without awareness in a “trance-like state” (9), rather than the focused one which is performed consciously and intentionally.

## Materials and Methods

### Participants

The initial study sample consisted of 600 participants aged from 18 to 63 years old (*M* = 23.88, SD = 6.88, 76.50% women, 22.83% men, 0.50% diverse) recruited from the general population through convenience sampling between July 2019 and December 2020 (a large part of the sample was recruited during the COVID-19 pandemic). Responders were invited to participation via advertisements placed on websites and social media applications. The participants who reported suffering from dermatological illnesses (*N* = 52, 8.67% of the total sample) were excluded from the study. The final sample was comprised of 546 subjects ranging in age from 18 to 60 (*M* = 23.92, SD = 6.87, 76.20% women and 23.80% men). Among these 375 (68.70%) participated in the study during the pandemic. All of the participants were Caucasians. Detailed characteristics of the study sample are presented in Table 1. There was no missing data. The Research Ethics Committee at the Institute of Psychology, Jagiellonian University reviewed and approved all the study procedures (KE/022018).

**Table 1:** Sociodemographic characteristics of the participants by pre-pandemic/pandemic subgroups.

	**Group: pre-pandemic (*****N*** **= 171)**			**Group: pandemic (*****N*** **= 375)**			**Group: all (*****N*** **= 546)**		
**Category**	***N*** **(Total)**	***N*** **(Women/men)**	**% of group**	***N*** **(Total)**	***N*** **(Women/men)**	**% of group**	***N*** **(Total)**	***N*** **(Women/men)**	**% of group**
**Employment**									
Employed	72	52/20	43.27	167	117/50	44.53	239	169/70	43.77
In education	124	96/28	72.51	320	247/73	85.33	444	343/101	81.31
Unemployed	1	1/0	0.58	3	3/0	0.80	4	4/0	0.73
**Place of origin**									
Rural area	44	37/7	25.73	151	118/33	40.27	195	155/40	35.71
<20,000 inhabitants	21	11/9	12.28	47	33/14	12.53	67	44/23	12.27
20,000–100,000 inhabitants	40	29/12	23.39	60	48/12	16.00	101	77/24	18.50
>100,000 inhabitants	66	51/15	38.60	117	89/28	31.20	183	140/43	33.52
**Marital status**									
Married/partnered	97	70/27	56.73	205	158/47	54.67	302	228/74	55.31
Single	74	58/16	43.27	170	130/40	45.33	244	188/56	44.69
**Experienced trauma**									
Any form of trauma	160	121/39	93.57	346	269/77	92.23	506	390/116	92.67
Emotional neglect	86	66/20	50.29	216	173/43	57.87	302	239/63	55.31
Emotional abuse	112	85/27	65.50	227	182/45	60.53	339	267/72	62.09
Bodily threat	105	81/24	61.40	217	166/51	57.87	322	247/75	58.97
Sexual harassment	24	22/2	14.04	62	54/8	16.53	86	76/10	15.75
Sexual abuse	21	18/3	12.28	40	35/5	10.67	61	53/8	11.17

### Measurements

#### The Traumatic Events Checklist (TEC)

The TEC is a self-report questionnaire composed of 29 items capturing five subscales: (1) emotional abuse (defined as being belittled, teased, called names, threatened verbally, or unjustly punished by parents, brothers and sisters); (2) emotional neglect (defined as being left alone or having received insufficient affection from parents); (3) bodily threat (being hit, tortured, or wounded by parents, brothers, or sisters; situations of receiving a threat to life from another person, e.g., during a crime); (4) sexual harassment (acts of a sexual nature that do not involve physical contact by parents, brothers, or sisters); (5) sexual abuse (unwanted sexual acts involving physical contact by parents, brothers, or sisters) (52). Participants report whether they have experienced individual traumatic life event by marking Yes/No answers. The overall score is obtained by summing up all item scores. A cumulative score can also be calculated. For confirmed items they also assess the duration of a particular traumatic event (in years), as well as its subjective impact (on a 5-point scale). In the analysis we take into account only the number of traumatic events reported by participants. The Polish version of the TEC was used in the study, the Cronbach's alpha calculated for the total score in the study sample was 0.72.

#### The Milwaukee Inventory for the Dimensions of Adult Skin Picking (MIDAS)

The MIDAS is a 12-item, self-administered measure developed to assess two subtypes of skin-picking behaviours: focused skin-picking (6-items) and automatic skin-picking (6-items). Each item is rated from 1 (not true for any of my behaviours of skin-picking) to 5 (true for all my behaviours of skin-picking). The Polish translation of the MIDAS was used in the current study. The Cronbach's alphas calculated for the study sample were: α = 0.90 for the focused skin-picking dimension, α = 0.85 for the automatic skin-picking dimension.

#### The Dissociative Experiences Scale-PL (DES-PL)

The DES-PL is a self-administered tool designed to measure the level of dissociation conceptualised as a continuum between normal experiences and pathological psychoform symptoms. It is composed of 28 items assessing a wide range of dissociative phenomena. Each item is answered on an eight-point scale, ranging from 0 (never) to 7 (once a day or more), measuring the frequency with which a participant experiences each of the dissociative-related phenomena. The overall score is obtained by summing up all the item scores. The DES-PL was found to have good psychometric properties (53). The Cronbach alpha calculated for the study sample was 0.93, and it was the same as previously reported (53).

#### Diagnostic Criteria for Skin-Picking Disorder

The participants were also provided a set of questions related to the DSM-5 diagnostic criteria of excoriation (skin-picking) disorder (1). They were asked: (1) if they picked the skin to such an extent that it results in noticeable skin damage? (2) if they made attempts to decrease or stop picking? (3) if skin-picking caused clinically significant distress or impairment in social, occupational, or other important areas of functioning; (4) if they suffered from a psychiatric or dermatological illness which caused picking? The possible answers were Yes/No.

#### Sociodemographic Data Sheet

The sociodemographic data sheet that was filled out by the participants included questions about their age, sex, marital status, employment and place of origin.

### Procedure

The research was conducted online. Before attending the study all participants were provided short, written information concerning the aim and length of the study. The information that participation was anonymous and that the obtained results would only be used for research purposes was also displayed to responders on the first sheet of the online study form. Subsequently, individuals who were interested in participating in the survey were asked to consent to participation by marking the appropriate box on the computerised study form. Next, they filled the survey online. No financial remuneration was offered to the participants. This study was part of a bigger project in which the data about personality, attentional control and coping strategies were also collected from the sample. Because of the limited scope of the article, the analysis of the interplay between those variables will be described in a separate publication.

## Results

### Data Analysis Plan

Firstly, the assumptions of univariate and multivariate normality and linearity were evaluated. The linear relationship between variables was confirmed using scatter plots. The assessment of distributions and joint multivariate kurtosis suggested non-normality—therefore tests and estimators that do not assume normality were utilised. Moreover, in case of the path model, bootstrap parameter estimates, confidence intervals, and significance levels were computed and reported. Zero-order Spearman correlations were calculated to assess relationship between variables. To determine whether there is a significant difference between groups of participants tested before and during the COVID-19 pandemic and between skin-picking and non-skin-picking groups the Mann-Whitney U test was used. The proportion of men and women, people who experienced trauma and those who did not, as well as people meeting the DSM-5 criteria of excoriation disorder and not meeting the criteria in groups was compared using chi-square test. The mediation model was tested by conducting a path analysis. Finally, to test if the relationships within the model are similar across conditions in which participants were tested (before vs. during the pandemic), multi-group path analysis was conducted. There was no missing data. All of the analyses were conducted using SPSS v. 26 and AMOS v. 26.

### Preliminary Analyses

Among 546 participants taking part in the study 268 (52.38%) reported that they pick the skin at least occasionally; 68 (12.45%) declared that they met the criteria of excoriation disorder according to DSM-5 (1). The individuals reporting picking were significantly younger than those who denied picking (22.88 vs. 24.92 years old): *U*(*N*_skin−picking_ = 268, *N*_control_ = 278) = 30142.00, *z* = −3.89; there was also predominance of women among those who pick the skin: χ2_(1)_ = 4.72, *p* = 0.03.

Correlation analysis showed that both types of skin-picking were related significantly and positively with dissociative tendencies as well as traumatic experiences. The only exception was sexual abuse that did not show significant relationship with both types of skin-picking. As expected, there was positive correlation between traumatic life events and dissociative experiences. Descriptive statistics and the results of correlation analysis are presented in Table 2.

**Table 2:** Descriptive statistics and correlation coefficients (Speraman's rho).

**Variable**	**Mean (SD)**	**Min/max**	**Skewness (SE)**	**Kurtosis**	**(1)**	**(2)**	**(3)**	**(4)**	**(5)**	**(6)**	**(7)**	**(8)**	**(9)**	**(10)**
Skin-picking - focused (1)	6.12 (6.98)	0/24	0.71 (0.11)	−0.85 (0.21)	1									
Skin-picking -automatic (2)	11.00 (4.75)	0/24	1.02 (0.11)	0.11 (0.21)	0.89^***^	1								
Dissociative experiences (3)	71.10 (27.80)	28/181	1.09 (0.11)	1.00 (0.21)	0.33^***^	0.32^***^	1							
Traumatic life events -total (4)	4.64 (3.31)	0/15	0.75 (0.11)	0.05 (0.21)	0.15^***^	0.16^***^	0.26^***^	1						
Emotional neglect (5)	0.86 (0.93)	0/3	0.76 (0.11)	−0.47 (0.21)	0.12^**^	0.11^**^	0.22^***^	0.68^***^	1					
Emotional abuse (6)	0.83 (0.78)	0/3	0.59 (0.11)	−0.30 (0.21)	0.17^***^	0.18^***^	0.19^***^	0.68^***^	0.44^***^	1				
Bodily threat (7)	0.91 (0.99)	0/6	1.24 (0.11)	2.01 (0.21)	0.12^**^	0.13^**^	0.20^***^	0.69^***^	0.32^***^	0.40^***^	1			
Sexual harassment (8)	0.17 (0.41)	0/2	2.31 (0.11)	4.69 (0.21)	0.09^*^	0.11^**^	0.10^*^	0.39^***^	0.21^***^	0.28^***^	0.27^***^	1		
Sexual abuse (9)	0.12 (0.36)	0/2	2.99 (0.11)	8.78 (0.21)	0.08	0.07	0.09^*^	0.33^***^	0.20^***^	0.21^***^	0.19^***^	0.41^***^	1	
Age (10)	23.90 (6.87)	18/60	2.63 (0.11)	7.47 (0.21)	−0.21^***^	−0.12^***^	−0.24^***^	0.05	−0.07	−0.02	0.09^*^	0.07	0.04	1
Gender^a^	-	-	-	-	0.13^*^	0.05	−0.03	0.06	0.08	0.07	−0.06	0.13	0.10	−0.09

* *p < 0.05*,

** *p < 0.01*,

*** *p < 0.001*,

a *point-biserial correlation coefficients are reported in case of gender (0-men, 1-women)*.

The proportion of participants who experienced at least one traumatic event in the past was similar in the group that declared picking the skin and the group that denied it: χ2(1, *N* = 546)=0.29, *p* = 0.59. However, participants who picked the skin experienced significantly more traumatic events (*M* = 5.06) than participants who did not engage in such behaviour (*M* = 4.24): *U*(*N*_skin−picking_ =268, *N*_control_ = 278) = 31876.50, *z* = −2.93, *p* < 0.01.

Between-group comparisons showed that pre-pandemic and pandemic samples did not differ significantly in terms of the level of dissociative experiences: *U*(*N*_pre−pandemic_ = 171, *N*_pandemic_ = 375) = 30,852.50, *z* = −0.71, *p* = 0.48, automatic skin-picking: *U*(*N*_pre−pandemic_ = 171, *N*_pandemic_ = 375) = 29,756.50, *z* = −1.41, *p* = 0.16, focused skin-picking: *U*(*N*_pre−pandemic_ = 171, *N*_pandemic_ = 375) = 29,914.50, *z* = −1.32, *p* = 0.19, nor in the total number of traumatic life events experienced earlier: *U*(*N*_pre−pandemic_ = 171, *N*_pandemic_ = 375) = 31,663.00, *z* = −0.24, *p* = 0.81. Participants who took part in the study before pandemic onset (*M* = 25.31) were significantly older [*U*(*N*_pre−pandemic_ = 171, *N*_pandemic_ = 375) = 26862.50, *z* = −3.07, *p* < 0.01] than those participating during the pandemic (*M* = 23.29). Proportions of men vs. women: χ2(1, *N* = 546)=0.25, *p* = 0.62, single vs. married/partnered: χ2(1, *N* = 546) = 0.20, *p* = 0.65, as well as proportion of people meeting excoriation disorder criteria vs. not meeting those criteria, were comparable in both subsamples: χ2(1, *N* = 546) = 0.13, *p* = 0.72. In the pandemic subsample there were more participants declaring rural area as place of origin (40%) than in the pre-pandemic subsample (26%): χ2(3, *N* = 546) = 12.97, *p* < 0.01.

### Mediation Model

To test the mediation effect, we followed the procedure described by MacKinnon (54), which requires the following three conditions to be met: (a) a significant association between traumatic life events and types of skin-picking; (b) a significant association between dissociative experiences and types of skin-picking while controlling for traumatic life events; and (c) a significant coefficient for the indirect path between traumatic life events and skin-picking via dissociative experiences. We used bias-corrected bootstrap 95% confidence intervals to determine whether the last criterion was met (55). Asymptotically distribution free estimation was implemented in the path analysis. Gender and age were controlled for. As gender was a categorical variable—dummy coding was used to code its values (male sex was coded as 0 and treated as reference variable). The model was just identified (zero degrees of freedom) so the model fit could not be assessed.

**Figure 1:**
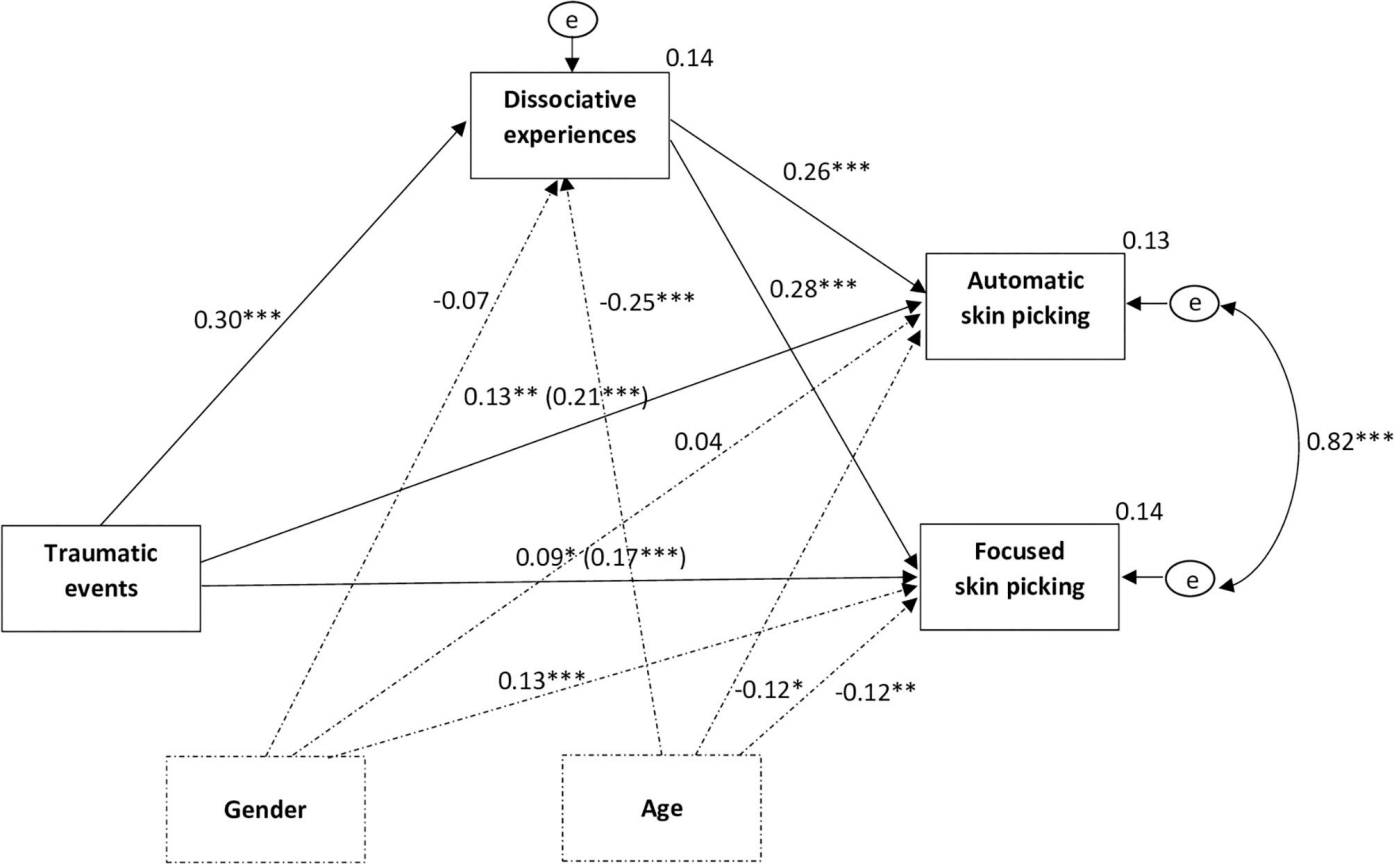
Dissociative experiences mediate the relationship between traumatic events and automatic and focused skin-picking. *N* = 594, *p < 0.05, **p < 0.01, ***p < 0.001; gender and age were controlled for in the analysis; exogenous variables were allowed to covary; the effects on the direct path from traumatic events to automatic and focused skin-picking depict the direct effect and the (total effect) accordingly; indirect effect of traumatic life events on automatic skin-picking via dissociative tendencies: β = 0.08; CI: 0.05 to CI = 0.11, *p* < 0.001; indirect effect of traumatic life events on focused skin-picking via dissociative tendencies: β = 0.09; 95%CI:0.05 to 0.13, *p* < 0.001.

In a model including only the direct path from traumatic life events to the types of skin-picking (and control variables), traumatic life events turned out to be significantly and positively associated with both types of skin-picking (automatic: β = 0.21, 95%*CI*: 0.13 to 0.27, *p* < 0.001; focused: β = 0.17, 95%CI: 0.09 to 0.25, *p* < 0.001). In a model containing the mediator, traumatic life events were positively and significantly associated with dissociative experiences: β = 0.30, 95%CI: 0.22 to 0.38, *p* < 0.001, which in turn were significantly and positively associated with both types of skin-picking (automatic: β = 0.26, 95%CI: 0.19 to 0.34, *p* < 0.001; focused: β = 0.28, 95%CI: 0.20 to 0.37, *p* < 0.001). The direct effects of traumatic experiences on automatic (β = 0.13, 95%*CI*: 0.05 to 0.20, *p* < 0.01) and focused (β = 0.09, 95%CI: 0.01 to 0.16, *p* < 0.05) skin-picking were still significant, although weaker. The indirect effect of traumatic life events on automatic and focused skin-picking through dissociative experiences was positive and significant (automatic: β = 0.08; 95%CI: 0.05 to UCI = 0.11, *p* < 0.001; focused: β = 0.09; 95%CI: 0.05 to 0.13, *p* < 0.001) suggesting partial mediation. The path model is presented in Figure 1.

### Multi-Group Analysis

Given that exposition to stressors related to current pandemic may affect both dissociation and skin-picking behaviours, the current study further examined whether the mediating effect of dissociation in the relationship between traumatic events and skin-picking types is consistent across the two study's conditions (pre-pandemic vs. pandemic). Multigroup analysis was used (56). Firstly, the unconstrained model (in which the structural weights were freely estimated across groups) was estimated. Secondly, the constrained model was estimated by constraining all of the structural weights to be equal between the two groups. Finally, the χ2 values and degrees of freedom of the unconstrained and constrained models were compared to determine if the models are significantly different (57). The chi-square difference test showed that the difference between constrained and unconstrained mediation model is not significant: χ2 diff (11) = 7.22, *p* = 0.78. The results of chi-square difference tests as well as bootstrap confidence intervals for each path of the model is presented in Table 3.

**Table 3:** Multi-group analysis: difference between paths in mediation model.

			**Bootstrap (***N*** = 2,000)**				
**Path name**	**β pre-pandemic**	**β pandemic**	**β diff**	**CI_Lower_95%**	**CI_Upper_95%**	***P***	**χ2 diff**
Traumatic life events -> dissociative experiences	0.28^***^	0.31^***^	−0.03	−0.20	0.15	0.77	χ2 diff (1) =0.09, *p* = 0.76
Traumatic life events -> focused skin-picking	−0.03	0.14^;**^	−0.16	−0.34	0.02	0.08	χ2 diff (1) =3.99, *p* = 0.07
Dissociative experiences->focused skin-picking	0.34^***^	0.27^***^	0.07	−0.13	0.28	0.51	χ2 diff (1) =0.51, *p* = 0.47
Traumatic life events->automatic skin-picking	0.05	0.16^;**^	−0.11	−0.29	0.07	0.27	χ2 diff (1) = 1.49, *p* = 0.22
Dissociative experiences->automatic skin-picking	0.31^;**^	0.25^^***^^	0.06	−0.15	0.27	0.60	χ2 diff (1) = 0.33, *p* = 0.56

** *p < 0.01,*

*** *p < 0.001*.

Additionally, the bootstrap procedure (*N* = 2000) was used to test the difference between indirect effects in both groups which also turned out to be insignificant (difference in standardised estimates for automatic skin-picking = 0.01, 95%CI: −0.07 to 0.10, *p* = 0.79; difference in estimates for focused skin-picking = −0.01, 95%CI: −0.06 to 0.11, *p* = 0.71).

## Discussion

In the current study we investigated the relationship between trauma experience and two types of skin-picking behaviours varying in terms of awareness. We also examined whether dissociative symptoms, being some of the most prominent trauma consequences, mediate the relationship between past adverse events and unconscious (so called automatic) style of skin-picking.

Regarding skin-picking prevalence we found that 52.38% of participants confirmed that they pick the skin at least occasionally; 12.45% gave a positive answer to the questions concerning excoriation (skin-picking) disorder diagnostic criteria, suggesting that their picking is of clinical relevance. These results indicate higher prevalence of both non-clinical and clinical skin-picking than in the prior study on excoriation spread in the Polish community sample (12). However, these discrepancies between studies may be explained by the difference in recruitment procedure. The current study was conducted online, therefore only individuals whose attention was captured by the advertisement and who accepted the invitation to participate fulfilled the study form, which may result in overrepresentation of skin-picking sufferers. In other words, responders who experience distress and problems in daily functioning due to their picking may have been particularly motivated to voluntarily participate in such a study.

In the current study we observed women's predominance among individuals reporting skin-picking. This finding seems to be inconsistent with the results of previous studies showing equal sex proportion in non-clinical samples (10, 13, 58). However, since over 12% individuals in this group defined themselves as meeting the diagnostic criteria of excoriation disorder, it is not surprising that we found a higher female ratio, usually observed in clinical samples (48, 59). The analyses also yielded the age difference between individuals reporting skin-picking and those who denied picking, with the “skin-picking” group being significantly younger. Age also negatively correlated with skin-picking intensity, both focused and automatic. This finding is in line with the observation that high rates of pathological skin manipulation are often reported by adolescents and young adults, which probably reflects the appearance of facial acne in this age (48, 60). People in early adulthood also experience greater intensity of negative emotions than older people (61, 62).

The main aim of our study was to examine the relationship between traumatic life events and skin-picking. In accordance with our assumption we found that both picking styles are weakly yet significantly associated with almost all types of adverse life events examined in the study, except for sexual abuse. What is more, the average number of traumatic events reported by individuals was significantly higher in the “skin-picking” group than in the “non-skin-picking” one. It suggests that, similarly as has been found in the case of trichotillomania, the experience of trauma may be listed among factors favouring skin-picking appearance, however, if we consider the relative strength of this relationship, this association seems to be not direct, and probably narrowing to individuals who are particularly prone to such symptoms due to genetic susceptibility or neurological impairments. It is plausible that in people without the OCD-spectrum vulnerability trauma elicits other forms of psychopathology, resulting in a wide range of consequences which have already been broadly described (22–27). In line with this interpretation, in the present study we found that in both “skin-picking” and “non-skin-picking” groups, the percent of people who experienced a traumatic event was similar. However, given the finding that individuals from the “skin-picking” group reported a higher average number of traumas than those who denied picking, it is also possible that skin-picking development is based on a cumulative effect of multiple exposure to several traumatic events.

The current study yielded that sexual abuse is the one type of traumatic events not related to skin-picking irrespective of picking styles. Although numerous previous studies showed the link between sexual abuse and psychopathological symptoms [e.g., (63–66)] they were mostly carried out in clinical samples and focused on individuals who developed full-blown disorders. On the contrary, our study was conducted in a non-clinical sample and most of the participants who reported skin-picking declared that they did not meet all of the DSM criteria for SPD. Moreover, only 11% of the total sample reported they experienced sexual abuse in the past and it was the least frequently reported type of trauma in the current study. Interestingly, the previous meta-analysis of the 43 studies exploring the link between sexual abuse and non-suicidal self-injurious behaviours (67), also showed very weak relationship between these two variables, especially in non-clinical samples. Furthermore, sexual abuse explained little or no unique variance in self-injurious behaviour when other risk factors were controlled for. Further studies on clinical groups are needed to examine whether clinically relevant skin-picking is related to sexual abuse to a greater extent than in a non-clinical one.

In line with our hypothesis we found a significant association between dissociative symptoms and automatic skin-picking. However, contrary to our expectations an almost identical relationship was observed in case of focused (conscious) skin-picking. It suggests that pathological skin manipulation is linked with dissociative tendencies regardless of the degree of picking awareness. Hence, the present data are consisted with the previous observation that, in general, the act of picking is associated with a feeling of trance or a feeling of being mesmerised (4, 48). However, our study also supplemented the prior observations through showing that dissociative symptoms explain a similar proportion of variance of both types of skin-picking.

It is likely that dissociative symptoms affect automatic and focused skin manipulation in different ways. In the case of automatic picking dissociation may be manifested primarily by the lack of awareness of picking activity, whereas in case of focused picking it can be expressed through inability to stop picking experienced subjectively as a “trance-like” state, however, with preserved awareness.

Our main goal was to investigate the mediation effect of dissociative symptoms on the relationship between trauma and automatic skin-picking in order to better understand the mechanism underlying the potential association between self-reported adverse life events and excoriation disorder. Our findings provided evidence that dissociative symptoms serve as a partial mediator not only in the relationship between trauma and automatic picking, but also in the link between trauma and focused picking style. The partial mediation effect obtained in the present study suggests that the nature of the trauma-picking connection is more complex and factors other than trauma-evoked dissociation are also involved in this relationship. For example, it has been hypothesised that skin-picking is related to disturbed activation and may serve as a method aimed at overactivation/underactivation adjusting (4, 18, 19). Since chronic overactivation is also listed among frequent trauma consequences it is likely that it constitutes another important factor linking traumatic events and skin-picking.

The comparison of data gathered during the COVID-19 pandemic and before pandemic onset did not yield significant differences in terms of both skin-picking and dissociative symptoms frequency. Additionally, conditions in which participants filled out the survey did not moderate any of the effects obtained in the study. This finding is in line with the recent longitudinal study showing that pandemic-related stress does not exaggerate skin-picking (5). However, it should be noted that during the pandemic most individuals from the general, non-clinical sample were able to continue their usual work activities via computers or other electronic devices despite the lockdown (5). What is more, the majority of them stayed at home with family members, therefore, they still had access to social support and could effectively regulate activation levels despite the limited possibility of leaving home.

There are several limitations associated with the current research. Firstly, in the study we used self-report tools to assess the severity of skin-picking, which may have resulted in inadequate estimations of picking behaviours in the study sample. In particular, the rates of automatic skin-picking may not reflect its actual symptoms prevalence due to limited awareness of such picking episodes. Also, the data concerning traumatic life events were provided via self-reports and were retrospective, therefore, they may have also been distorted. Secondly, our dataset was limited to the data provided online which limited the study sample to the individuals whose attention was captured by the advertisement due to their skin-picking behaviours. This methodological issue might have caused overrepresentation of skin-picking sufferers in the study sample. Thirdly, cross-sectional design of the study precludes causal inferences. Further longitudinal studies are warranted to confirm whether psychological trauma and dissociative experiences may affect the development of skin-picking. What is more, our findings are not generalizable beyond a non-clinical sample. Further research will have to investigate to what extent the mediation model confirmed in the non-clinical group concerns clinically relevant skin-picking and clinical forms of dissociation.

Overall, our study provides support for the hypothesised relationship between trauma, dissociation and skin-picking behaviours in a non-clinical sample. We found that skin-picking is related to various types of traumatic events (emotional neglect, emotional abuse, bodily threat, sexual harassment), and that dissociative symptoms partially mediate this relationship. Therefore, our results tentatively suggest that trauma may be of importance in the onset of skin-picking in the non-clinical population and may affect picking through dissociative symptoms. What is more, we also demonstrated that the mediation effect appears regardless of the skin-picking style, that is, may be observed not only in case of unaware “automatic” picking, but also in case of intentional “focused” skin manipulation.

This study may have potential implications for the clinical practise. It suggests, that psychotherapy, focused on working through memories and emotions tied to traumatic experiences, may be beneficial for some of the patients suffering from excoriation disorder. Additionally, increasing person capacity to tolerate and down-regulate negative affective states by teaching him/ her more adaptive methods of distress reduction may be important target of therapeutic interventions aimed at skin picking behaviours. However, further longitudinal studies are required to verify if the mediation model is applicable in the clinical context.

## Data Availability Statement

The raw data supporting the conclusions of this article will be made available by the authors, without undue reservation.

## Ethics Statement

The studies involving human participants were reviewed and approved by The Jagiellonian University Institute of Psychology Research Ethics Committee. The patients/participants provided their written informed consent to participate in this study.

## Author Contributions

JK and KP designed the study and interpreted the data. KP wrote the manuscript. JK ran the analyses and edited manuscript. RA-R and AK-W gathered the data. All authors approved the final version of the manuscript.

## Conflict of Interest

The authors declare that the research was conducted in the absence of any commercial or financial relationships that could be construed as a potential conflict of interest.
